# Errors and Their Mitigation at the Kirchhoff-Law-Johnson-Noise Secure Key Exchange

**DOI:** 10.1371/journal.pone.0081103

**Published:** 2013-11-26

**Authors:** Yessica Saez, Laszlo B. Kish

**Affiliations:** Department of Electrical and Computer Engineering, Texas A&M University, College Station, Texas, United States of America; Universita' del Piemonte Orientale, Italy

## Abstract

A method to quantify the error probability at the Kirchhoff-law-Johnson-noise (KLJN) secure key exchange is introduced. The types of errors due to statistical inaccuracies in noise voltage measurements are classified and the error probability is calculated. The most interesting finding is that the error probability decays exponentially with the duration of the time window of single bit exchange. The results indicate that it is feasible to have so small error probabilities of the exchanged bits that error correction algorithms are not required. The results are demonstrated with practical considerations.

## Introduction

### 1.1 The KLJN secure key exchange

In today’s era, network security has become one of the most important aspects in everyday life. Whether it is a large, small, private, or a government organization, it is very important to focus on security, especially when the data being sent, received, or stored contain confidential, sensitive information, such as personal information.

In private-key based secure communication, the two communicating parties (Alice and Bob) generate and share a secure key, which is typically represented by a random bit sequence. It is important to note that the security of a communication cannot be better than the security of the exchange of the key it uses. During this key exchange, the eavesdropper (often referred to as Eve) is continuously monitoring the related data. In today’s Internet-based secure communications, typically a software–based key generation and distribution is utilized. However, in this method the whole information about the secure key is publicly available [1] and Eve’s access to this information is limited only by her computational power. In other words, this method provides only a (*computationally*) *conditional* security level, which represents a non-future-proof-security [2]–[4]. It means that with a sufficiently enhanced computation power or an efficient future algorithm, Eve may be able to crack the key and all the information in the communication may become accessible.

Therefore, scientists and researchers have been working on exploring proper laws of physics to find new key exchange schemes where the information that can be measured by Eve is zero. Particularly, they have been exploring key exchange schemes where the amount of information extracted by Eve does not depend on her computational power. When the security measures are determined at Eve's maximal ability (limited only by the laws of physics and the protocols working conditions), that is referred as *unconditional security*, a term that is often interchanged with *information theoretic security* [1]. Information theoretic (unconditional) security can be *perfect* if Eve can extract no information, or *imperfect*, if Eve can extract only a small, commonly accepted amount of information. (This is allowed for practical purpose because this small information leak can further be decreased by privacy amplification, if the fidelity of the key exchange between Alice and Bob is good enough.) These terms are often misunderstood, and it is a frequent mistake in claims to misuse *unconditional security and imply perfect security* by that.

It is important to emphasize that the goal to generate/distribute a perfectly secure key is similar to approaching infinity. Perfectly secure key distribution of a key of finite length can never be reached with a real physical system within a finite duration of time. However, it is one of the goals of physical informatics to find out schemes that can arbitrarily approach (though never reach) perfect security [2].

The earliest and most famous scheme based on the laws of physics that is claiming unconditional security is the Quantum Key Distribution (QKD) [5]. The information theoretic security of this scheme is usually based on the assumption that Eve's actions will disturb the system (in accordance with the theory of quantum measurements and the no-cloning theorem) and cause errors, which uncover the eavesdropping. Note, there are some promising non-QKD initiatives that involve new types of quantum effects [6], [7].

At the fundamental side, there are ongoing debates between experts about the reachable levels of security in QKD [8]–[12]. At the practical side, there are some issues associated with this scheme, such as range, price, and robustness. Moreover, it is interesting to note that recently all the commercial QKD devices and many laboratory devices have been cracked by quantum-hacking [13]–[27]. While most of these practical weaknesses seem to be design flaws, not fundamental security problems; they still mean that current practical QKD has yet conditional security: the conditions are that Eve is not knowledgeable enough or she does not have the proper hardware to utilize the design flaws for an attack. The impressive list of papers [13]–[27] shows that there are enough knowledgeable Eves out with sufficient resources at the moment.

Until 2005 QKD was the only accepted scheme that was able to offer a key exchange with information theoretic security in the ideal (mathematical) situation. In 2005, the Kirchhoff-Law-Johnson-(like)-Noise (KLJN) secure key distribution was introduced [28], where the term "totally secure" was used instead of the correct "perfectly secure" expression. Later (2006), the KLJN system had been built and demonstrated [29]. KLJN is also a key exchange scheme with information theoretic security [3] and it is based on Kirchhoff’s Loop Law of quasi-static electrodynamics and the Fluctuation and Dissipation theorem of statistical physics. Its security against passive attacks is ultimately based on the Second Law of Thermodynamics [28], which means that it is as hard to crack the key exchange as to build a perpetual motion machine (of the second kind). At practical conditions it uses enhanced (electronically generated) Johnson noise with high noise temperature, where quasi-static and thermodynamic aspects must be emulated as exactly as possible in order to approach perfect security.

First, we present a brief description (based on [2]–[4], [28]) of the working principle of the KLJN system. The core KLJN system, without the defense circuitry against invasive attacks and vulnerabilities represented by non-ideal building elements is shown in the following figure.

**Figure 1:**
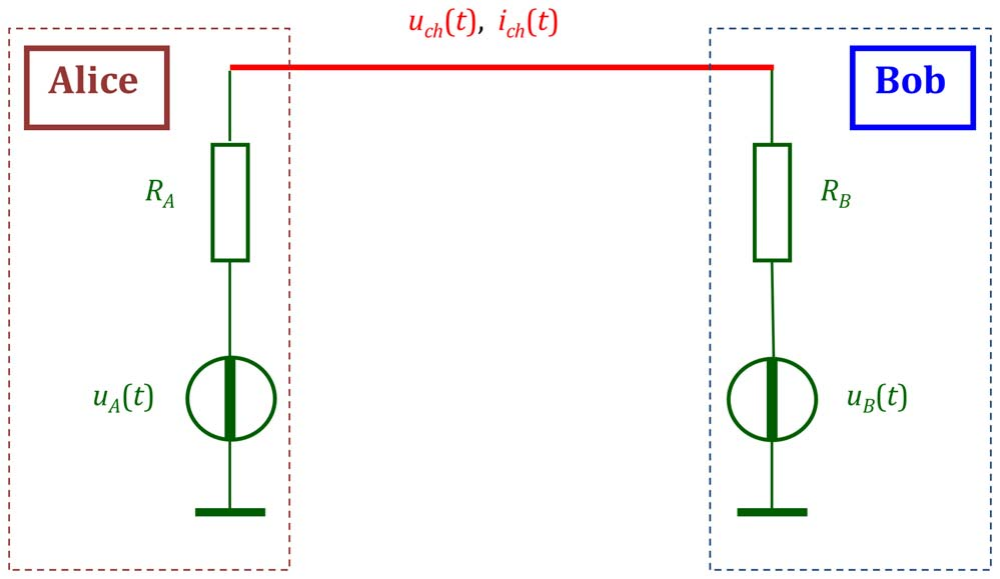
Outline of the core KLJN secure exchange scheme [2]–[4,28] without the defense elements against active (invasive) attacks or attacks utilizing non-ideal components and conditions. 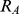
, 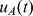, 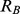, and 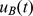 are the resistor values and noise voltages at Alice and Bob, respectively. 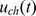 and 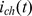 are channel noise voltage and current, respectively.

The core KLJN channel, see Fig. 1, is a wire line to which Alice and Bob connect randomly selected resistors 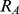 and 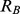, respectively, where 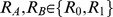. 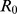 represents the low (0) bit and 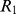 the high (1) bit, respectively [28]. At the beginning of each bit exchange period, BEP, (also called KLJN clock period), Alice and Bob, who possess identical pairs of the resistors 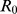 and 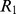, randomly select and connect one of these resistors. The Gaussian voltage noise generators represent either the Johnson noises of the resistors or external noise generators delivering band-limited white noise with publicly known bandwidth and effective noise temperature 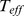 [2], [3], [28], [29]. The noise voltages of Alice and Bob are 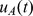 and 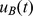, respectively, where 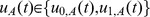 and 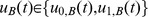 yield a channel noise voltage 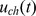 between the wire line and the ground and a channel noise current 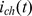 in the wire.

Alice and Bob measure the mean-square noise voltage and/or current amplitudes, that is 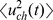
and/or 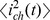, within the BEP in the line. Thus, by applying Johnson’s noise formula and Kirchhoff’s loop law the theoretical prediction is that the mean-square noise voltage and current (i.e. the integral of the corresponding power spectral densities [2], [28]) for a given channel noise bandwidth 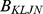 and temperature 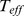
are given as follows:



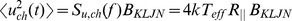


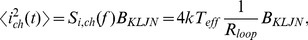
(1)


where 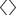 represents ideal (infinite-time) time average, 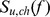 is the power density spectrum of channel voltage noise, 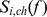 is the power density spectrum of channel current noise, 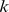 is the Boltzmann constant, 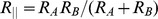 and 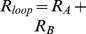.

Ideally, by comparing the result of the accurate measurement of the mean-square channel voltage or current with the corresponding theoretical value in Eq. 1, the total loop resistance will be publicly known. Alice and Bob know their own resistor values and thus they can deduce that resistance value from the loop resistance to learn the resistance at the other end. Consequently, they can distill the actual bit value at the other side of the wire.

If Alice and Bob use the same resistance values, Eve can also recognize that bit situation because the total resistance is either the lowest or the highest value of the three possible resistance values. Thus, the resistor situations (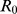,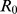) and (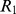,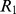) represent a non-secure bit exchange since Eve can also find out the resistors values, their exact locations, and the status of the bits. On the other hand, the cases (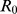,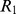) and (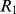,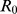), which yield identical mean-square noise in the line, represent a secure bit exchange situation because Eve is unable to locate the resistors, therefore, she cannot decide if Alice (and Bob) has a bit 1 or 0. This security is provided by the Second Law of Thermodynamics, which prohibits any directional information concerning the resistors at the two sides in thermal equilibrium [2], [28]. In other words, it is as difficult to extract these secure bits by Eve as to build a perpetual motion machine (of the second kind). In conclusion, on average, 50% of the bits can be kept because they are secure. The other 50% of the bits representing the non-secure situations is discarded by the protocol.

Note: the securely exchanged bits have opposite values at Alice and Bob, thus they must publicly agree which one of them will invert the exchanged bit to have identical keys at the two ends.

The fully armed KLJN system is secure even against the man-in-the-middle-attack [30]. One of the important potential applications [32] is to integrate the KLJN system on computer chips and provide unconditional security within computers and high-security instrumentations where the processors, hard drives, keyboards, etc. would secure their communications by keys shared via the KLJN protocol. Another, potential application is, at a much greater scale, to build a network of KLJN systems utilizing already existing wire lines [4], [33], [34], particularly, realizing and unconditionally secure "smart grid" [4] (advanced electrical power distribution network).

### 1.2 Known attack types

Below, based on [2], we briefly survey all the published attack types. Due to the simplicity of the KLJN system, there are very few attack types available. The method of comparing the instantaneous values of voltage and current at the two ends and discarding risky 01/10 bits [28], [30], [31] (not discussed here in details) protects against all these types of attacks. But even without discarding the risky bits, passive attacks by Eve utilizing non-idealities suffer from weak signal-to-noise ratio due to poor statistics, see below.

A practically unimportant but theoretically valid type of attack was shown by Hao [36] who pointed out that the non-ideal situation of different temperatures could separate the noise levels of the 01 and 10 bit situations, thus they could give out some information to Eve. In a response by Kish [37], it was pointed out that practical problems of accuracy do not challenge the conceptual security of ideal schemes and was estimated that, even at practical situations, the information leak is negligible due to this attack. Later, it was shown in the experimental paper of Mingesz et al. [29] that a modest 14-bit accuracy of temperatures (noise generators) practically prohibit Eve to extract any useful information (with information leak less than 10^−10^) by utilizing the Hao attack.

Scheuer and Yariv [38] analyzed the case of non-zero wire resistance where the mean-square voltages are different at the two ends in the case of the 01 and 10 bit situations. However, their calculation was incorrect including the physical units of some of the main results. Kish and Scheuer [39] carried out new, correct calculations and showed that the actual effect is about 1000 times weaker than predicted by Scheuer and Yariv. Earlier, Kish pointed out [37] in his response to [36] that at similar conditions Eve's statistic was very poor and the extracted information was practically miniscule even without the defense of discarding the risky bits. This claim was experimentally verified by Mingesz et al. [29], who showed that at clock period of 50 times of the noise correlation time, 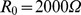, 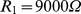, and wire resistance 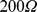, the information leak of exchanged raw bits to Eve was 0.19% while the fidelity between Alice and Bob was 99.98%. These results indicate that the key exchange has excellent fidelity even without error correction and that the security can be made reasonably good even without dropping the risky 01/10 bits (after current/voltage comparison at the two ends) and without privacy amplification [29].

Liu [41] used a cable simulator to evaluate the impact of delays and reflections on the security. He obtained the surprising results that, with the experimental parameters [6], Eve successfully guessed 70-80% of the key bits. In a critical study of Lui's simulations, Kish and Horvath [31] pointed out that the chosen wave impedances of the simulated cable to reach these results were unphysical: for example, a center wire diameter of 1 millimeter implies a coaxial cable with outer diameter of 28000 times greater than the size of the known universe.

Observing transients after switching the resistors has been mentioned as a potential source of information leak; however, so far they have never been utilized. During the experimental studies, the noise was ramped up at the beginning of the clock period and ramped down at the end, thus the switching of resistors took place when the voltage and currents were zero in the line.

Note, a fully transient-free protocol is described in a recent work [48].

According to [40], one of the most efficient attack types would be utilizing capacitive currents via the cable capacitance, though it has never been tested. Mingesz et al. [29] showed a hardware based defense "capacitance killer" against this attack. Ultimately, the method of discarding the risky bits after current/voltage comparison at the two ends [28], [30], [31] and/or, in the case of negligible error probability, privacy amplification [35] are the tools to approach perfect security.

### 1.3 Bit errors in the KLJN key exchange

Due to the finite duration 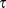 of the bit exchange period BEP, the measurement results of mean-square amplitudes have statistical inaccuracies. The duration 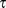 of the BEP must be long-enough compared to the correlation time of the noise (approximately the reciprocal noise-bandwidth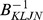) to achieve a satisfactory statistics and safely distinguish between the different resistor situations. Still, with a low probability, these uncertainties can trigger a bit error.

In the experimental demonstration Mingesz et al. [29] were able to optimize the system to have a fidelity of 99.98% (error probability 0.02%) however no mathematical analysis or design tools have been shown to address this problem. Therefore, our goal in this paper is to classify the different types of bit errors in the ideal KLJN system and analyze their impact.

## Discussion and Results

### 2.1 KLJN Errors

In this "startup" paper about error analysis, we assume the ideal situation of the KLJN system where all the non-ideal features of real systems are neglected. The error analysis of non-ideal systems will be done in future works.

**Figure 2:**
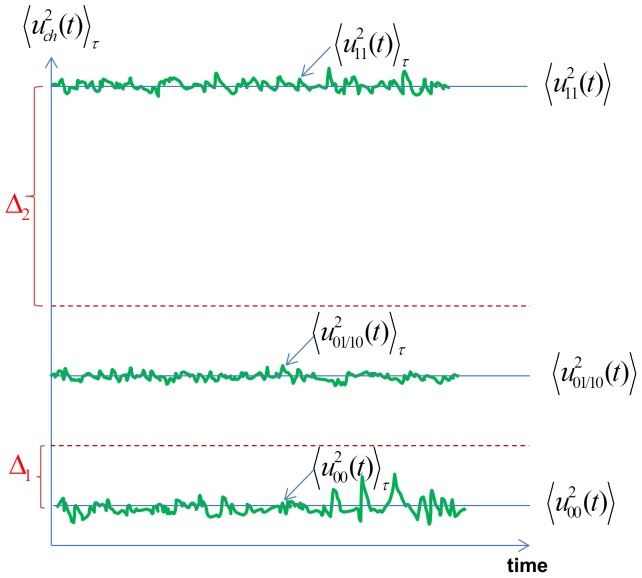
Illustration of the fluctuations of the finite-time mean-square voltage levels around their exact value and thresholds for interpretation (the scale is arbitrary). 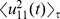
, 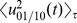, 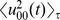 are the measured mean-square channel noise voltages at the 11, 01/10 and 00 bit situations, respectively. The solid lines with the quantities in 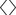 represent ideal (infinite-time) averages. For the sake of simplicity we assume 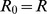 and, 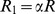 with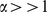.

Bit errors occur when the actual value of the mean-square noise results in an incorrect bit interpretation. Figure 2 represents the mean-square channel noise voltage levels, where 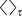 indicates finite (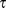) time average implying random fluctuations (statistical errors) around the real mean-square value.

The 11 bit situation (when Bob’s and Alice’s chosen resistors are 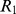 and their noise voltages are 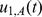 and 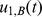, respectively) results in the mean-square channel noise voltage 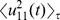. Similarly the 01/10 situations yield 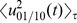 and the 00 bit arrangement results in 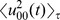. The threshold values 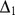 and 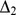 provide the boundaries to interpret the measured mean-square channel voltage over the 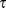 time window, see Fig. 2. The bit interpretation is 00 when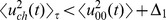, and 11 when 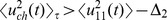. The secure bit situation 01/10is interpreted when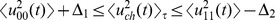.

An example for a bit error is the rare occurrence when the finite-time mean-square voltage of the 00 case, 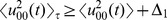, is interpreted as the 01/10 bit situation, which is incorrect and an example of a bit error.

The different types of errors are shown in Table 1.

**Table 1:** Types of errors in the KLJN bit exchange.

		Actual Situation		
		00	11	01/10
**Measurement Interpretation (Decision)**	00	Correct (no error)	Error, removed (automatically)	Error, removed (automatically)
	11	Error, removed (automatically)	Correct (no error)	Error, removed (automatically)
	1/10	Error* (probability?)	Error* (probability?)	Correct (no error)

* The rest of the paper addresses these errors and their probability.

Some of the errors situations, as shown in Table 1, are considered to be self-corrected by the protocol. This is because, as aforementioned, the 00 and 11 bit situations are discarded.

The rest of the paper is dealing with the analysis of errors indicated with * in Table 1.

### 2.2 Error probabilities in the KLJN scheme

Alice and Bob can calculate the total resistance in the system by measuring the mean-square noise voltage and/or current amplitudes, that is, 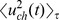 and/or 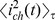. Below we evaluate the errors in the former case while the case of current-based evaluation can be done in a very similar fashion.

### 2.2.1 Error probability due to inaccuracies in noise voltage measurements. a) Probability of the 00  =  = > 01/10 type errors

Let 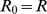 and 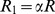 with 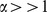. Note, the choice of 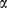 does not influence the resulting equations but it determines the upper limit at choosing the values of 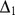 and 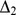 (see Eqs. 1). Then, the mean-square channel noise voltage for infinite-time average at the 00 bit situation is given as:


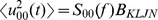
, (2)

where 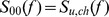 at the bit situation 00. Because 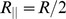, from Eqs. 1, we obtain:
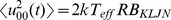
(3)


**Figure 3:**
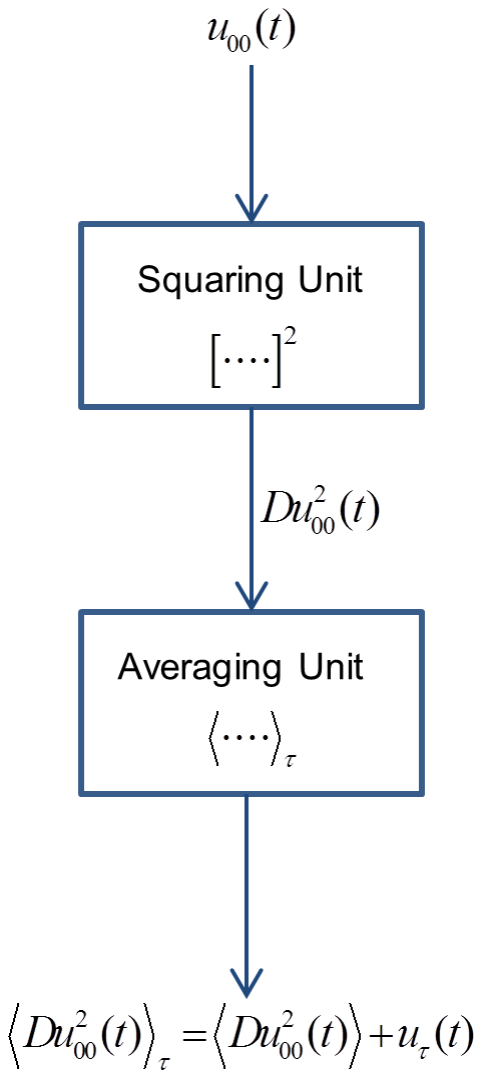
Illustration of the measurement process at 00. 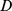
 is calibration coefficient of the squaring device to provide a Volt unit with the correct numerical value for the squaring operation.

During the BEP, only the duration 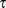 is available for Alice, Bob and Eve to determine the mean-square channel noise because, after that, a new bit exchange begins. The block diagram of the measurement process is shown in Fig. 3.

The channel voltage enters into a squaring unit. At its output, the signal is still voltage (because it is a voltage-signal-based electronics) and the numerical value of its instantaneous amplitude is equal to the square of the instantaneous amplitude of the input voltage. This fact is mathematically expressed by 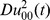, where 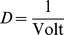 is the transfer coefficient of the device to provide a Volt unit also for the square [42]. After averaging for the finite-time 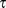 duration, the obtained measurement result is 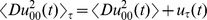, where the averaging can be represented by a low-pass filtering with cut-off frequency 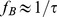.

While 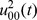 is not Gaussian, its finite-time average 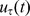 is Gaussian with high accuracy due to the Central Limit Theorem, because 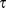 is much longer than the correlation time of the AC component 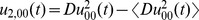 of 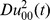, as 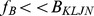. The probability of 00  =  = > 01/10 type errors is the probability that the AC component *remaining* after the finite-time average of 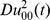 defined as 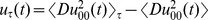 is beyond the threshold: 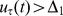. This can be evaluated by the error function, however, requires numerical integration.

To have an analytic formula, which is a good approximation and has the exact scaling in the small error probability limit, that is, when 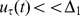 is satisfied, we can use Rice's formula [43], [44] of threshold crossing frequency, see similar solutions for estimating the probability of thermal noise induced switching errors [45]–[47]. The estimation of error probability is based on the fact that, in the small error limit, the probability of repeated threshold crossings within the correlation time of the band-limited noise converges to zero. The correlation time of 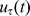 is also equal to 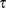 thus each threshold crossing (in a chosen but fixed direction) indicates an independent error. The ratio of the mean threshold crossing frequency 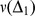 and 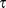 is a good estimation of the error probability in this limit [45], [46]. We compared the predictions of the Rice formula with the prediction based on numerically evaluated error function and found that the Rice formula gave always more pessimistic error estimation. The variation of the threshold resulted in changing the error probability prediction by the Rice formula and the error function by factors of ∼10^43^ and ∼10^44^, respectively. In the large error probability situation, the Rice formula predicted about 2 times greater error while, in the low error probability situation, about 18 times greater error. This is a negligible difference not only due to the 10^43^ – 10^44^ variation during the study but also because the exact error probability slightly depends on the fine details of the protocol not discussed here. To have analytic error estimation, we proceed as follows.

According to Rice, the mean frequency 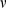 of crossing the level 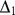 by a Gaussian with power density spectrum 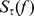 is given as:
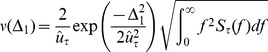
(4)


where 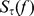 is the power density spectrum of 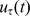 and 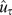 is its RMS value, 
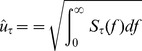
.

For normalization purposes, we choose the 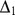 threshold level as a fraction of the *measured* mean-square channel noise, where the transfer coefficient *D* of the squaring unit is also taken into the account:



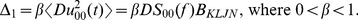
(5)




**Figure 4:**
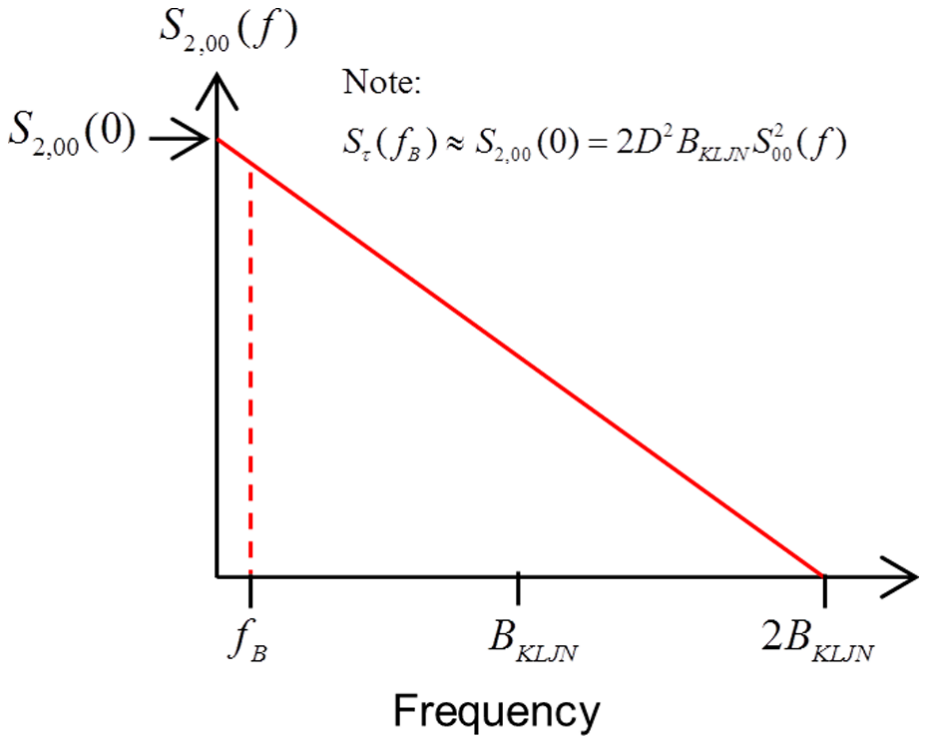
Power Spectral Density (PSD) of the product of two independent noises. 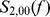
 is the power density spectrum of the AC component 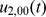 of the (non-averaged) 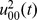.



According to [42], the power density spectrum, 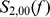, of the AC component 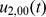 of the (non-averaged) 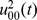 is given as (note typos of missing factor of 2 in Eqs. 6 and 7 in [42], see Fig. 4):

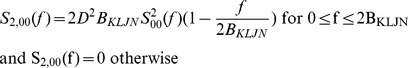
(6)


 


**Figure 5:**
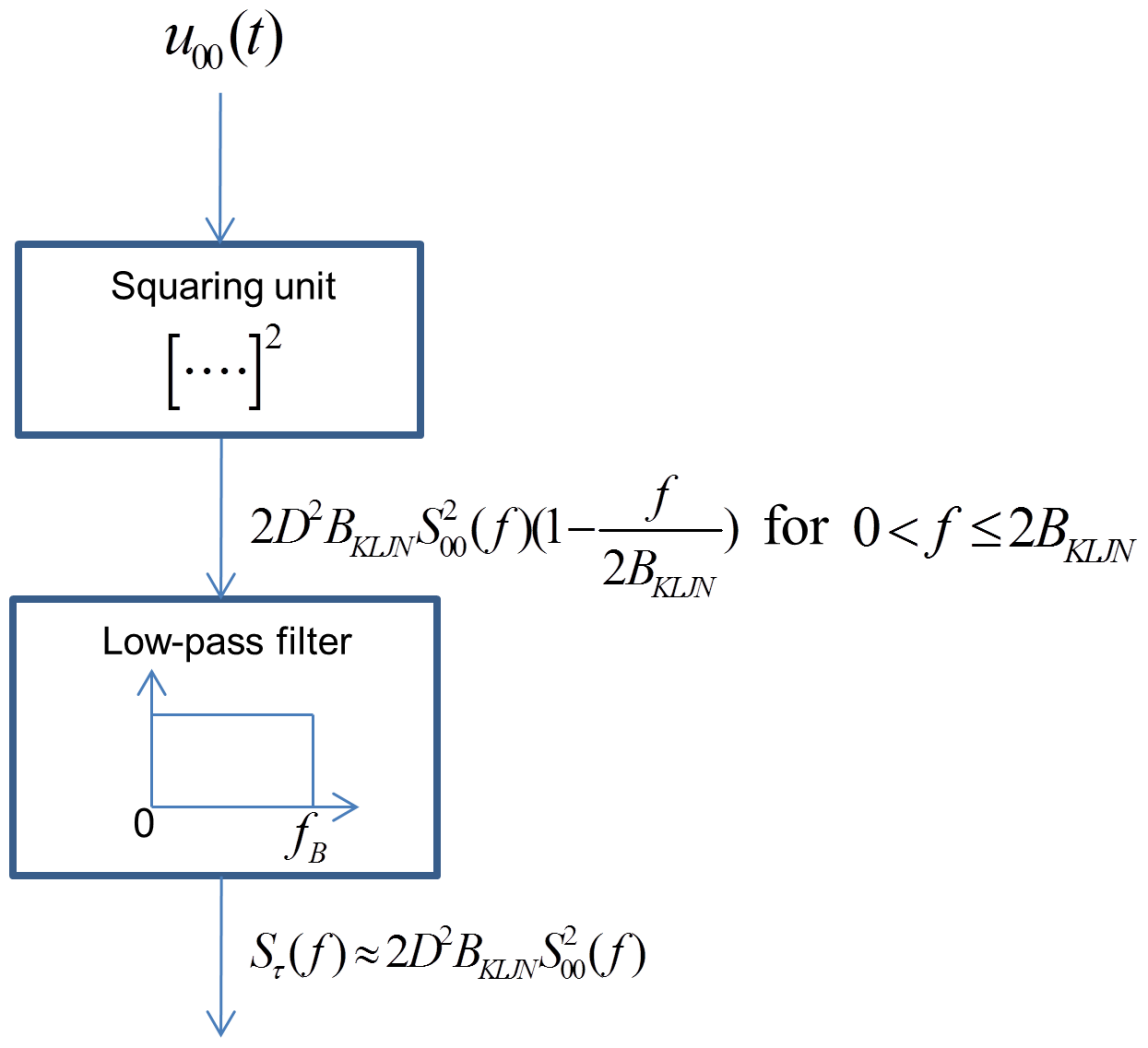
Spectra at the 00 bit situation. The low-pass filtering effect of the time averaging cuts off this spectrum for 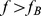 but keeps the 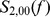 spectrum for 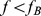. Since 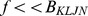, the value of 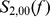 within the frequency band 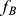 can be approximated by its maximum, so that 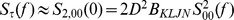.

The low-pass filtering effect of the time averaging cuts off this spectrum for 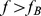 but keeps the 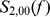 spectrum for 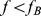. Because 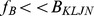, the value of 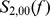 within the 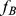 frequency band can be approximated by its maximum, 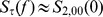. Figure 5 summarizes these findings.

Let us suppose that 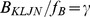. Then



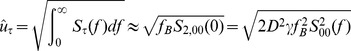
(7)


see text above and Figure 3 for explanation of the approximation. The frequency 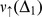 of unidirectional level crossings is half of the level crossing frequency predicted by the Rice formula:



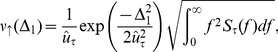
(8)


where
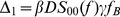
(9)


From Eqs. 7 and 9, we obtain
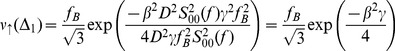
(10)


In the high threshold situation the errors follow a Poisson statistics, thus the error probability during a time interval is equal to the expected numbers of errors within this interval provided this number is much less than 1.

Thus the probability 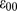 of 00 =  = >01/10 type of errors in the case of 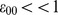 is:
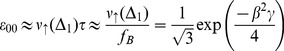
(11)


It is important to realize that the error probability is an exponential function of the parameters. The 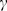
parameter (which is proportional to the length of time average) is particularly important because it is not limited in size.

### b) Probability of the 11  =  = > 01/10 type errors

We can follow the same procedure as above. Instead of 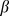 we introduce 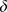 with similar meaning, see Fig. 2 and Eq. 5:



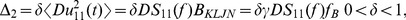
(12)




where 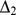 is the threshold for the 11 =  = >01/10 type errors and 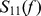 is the channel noise spectrum at the 11 bit situation.

The same type of calculations as given above yields the probability 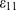 of 11 =  = >01/10 type errors:



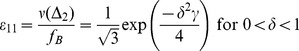
(13)




The error probability is again an exponential function of the parameters.

### 2.3 Illustration of the results with practical parameters

To demonstrate the results, we assign possible practical values to the parameters.

For 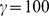 and 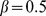 (a choice allowed due to the 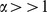 condition, see Eqs. 1) the bit error probability 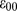 is:



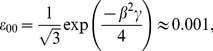
(14)


which is a value near to the experimental value (0.0002) obtained in [29] with the same 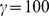 value (note the 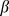 value is not available in [29] however the 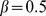 choice is a practical one).

If this value is too large, just by increasing the 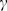 parameter (and the time average window 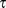) by a factor of 2, and in this way slowing down the bit exchange by the same factor, will result in the square of the above error probability value:



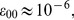
(15)


which is satisfactory for most applications. It is important to note that no error correction algorithm is used for this error reduction.

## Methods and Conclusions

We have classified and analyzed the types of errors of bit exchange between Alice and Bob in the KLJN secure key exchange. Some types of errors are automatically removed by the original protocol. We mathematically analyzed the error probabilities and their dependence on the KLJN parameters of the errors that are not removed by the protocol. We identified the important parameters and the results show that the error probability decays exponentially by increasing these parameters. The most important of such parameters is the duration 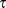 of key exchange because its value is not limited. The results indicate that it is reasonable to achieve error probabilities that are small enough to avoid the need for error correction algorithms.

Further open questions are how to combine current and voltage measurements to further reduce these errors and what is the error situation in the new advanced KLJN protocols proposed recently [48].

## References

[1] Liang Y, Poor HV, Shamai S (2008) Information theoretic security. Foundations Trends Commun. Inform. Theory 5: 355–580 10.1561/0100000036

[2] Mingesz R, Kish LB, Gingl Z, Granqvist CG, Wen H, et al (2013) Unconditional security by the laws of classical physics. Metrology & Measurement Systems 20: 3–16 DOI: 10.2478/mms-2013-0001 ; Mingesz R, Kish LB, Gingl Z, Granqvist CG, Wen H, et al. (2013) Information Theoretic Security by the laws of classical physics. In: Balas VE et al. (Eds.), Soft Computing Applications, AISC 195: 11–25. DOI: 10.2478/mms-2013-0001 10.2478/mms-2013-0001; Mingesz R, Kish LB, Gingl Z, Granqvist CG, Wen H, et al. (2013) Information Theoretic Security by the laws of classical physics. In: Balas VE et al. (Eds.), Soft Computing Applications, AISC 195: 11–25. DOI: 10.1007/978-3-642-33941-7_5

[3] Kish LB, Abbott D, Granqvist CG (2013) Critical analysis of the Bennett-Riedel attack on secure cryptographic key distributions via the Kirchhoff-law-Johnson-noise scheme. PLoS ONE in press. 10.1371/journal.pone.0081810 PMC3864860 24358129

[4] Gonzalez E, Kish LB, Balog R, Enjeti P (2013) Information theoretically secure, enhanced Johnson noise based key distribution over the smart grid with switched filters. PLoS ONE 8: e70206 10.1371/journal.pone.0070206 23936164 PMC3723689

[5] Bennett CH, Brassard G, Breidbart S, Wiesner S (1982). Quantum cryptography, or Unforgeable subway tokens. Advances in Cryptology: Proceedings of Crypto ’82, Santa Barbara, Plenum Press, pp. 267–275.

[6] Yuen HP (2009) Key Generation: Foundation and a New Quantum Approach, IEEE J. Sel. Top. . Quantum Electron. 15: 1630–1645 10.1109/JSTQE.2009.2025698

[7] Salih H, Li ZH, Al-Amri M, Zubairy H (2013) Protocol for direct counterfactual quantum communication. . Phys. Rev. Lett. 110: 170502 10.1103/PhysRevLett.110.170502 23679694

[8] Yuen HP (2012) On the Foundations of Quantum Key Distribution- Reply to Renner and Beyond. manuscript http://arxiv.org/abs/1210.2804.

[9] Yuen HP (2012) Unconditional Security in Quantum Key Distributions. manuscript http://arxiv.org/abs/1205.5065.

[10] Hirota O (2012) Incompleteness and Limit of Quantum Key Distribution Theory. manuscript http://arxiv.org/abs/1208.2106.

[11] Renner R (2012) Reply to Recent Scepticism about the Foundations of Quantum Cryptography. manuscript http://arxiv.org/abs/1209.2423.

[12] Yuen HP (2012) Security Significance of the Trace distance Criterion in Quantum Key Distribution. manuscript http://arxiv.org/abs/1109.2675.

[13] Merali Z (2009) Hackers blind quantum cryptographers. Nature News. DOI: 10.1038/news.2010.436.

[14] Gerhardt I, Liu Q, Lamas-Linares A, Skaar J, Kurtsiefer C, et al.. (2011) Full-field implementation of a perfect eavesdropper on a quantum cryptography system. Nat. Commun. 2. DOI: 10.1038/ncomms1348. 10.1038/ncomms1348 21673670

[15] Lydersen L, Wiechers C, Wittmann C, Elser D, Skaar J, et al (2010) Hacking commercial quantum cryptography systems by tailored bright illumination. Nat. Photonics 4: 686–689 10.1038/NPHOTON.2010.214

[16] Gerhardt I, Liu Q, Lamas-Linares A, Skaar J, Scarani V, et al (2011) Experimentally faking the violation of Bell's inequalities. Phys. Rev. Lett. 107: 170404 10.1103/PhysRevLett.107.170404 22107491

[17] Makarov V, Skaar J (2008) Faked states attack using detector efficiency mismatch on SARG04, phase-time, DPSK, and Ekert protocols. Quantum Information and Computation 8: 622–635.

[18] Wiechers C, Lydersen L, Wittmann C, Elser D, Skaar J, et al (2011) After-gate attack on a quantum cryptosystem. New J. Phys. 13: 013043 10.1088/1367-2630/13/1/013043

[19] Lydersen L, Wiechers C, Wittmann C, Elser D, Skaar J, et al (2010) Thermal blinding of gated detectors in quantum cryptography. Opt. Express 18: 27938–27954 10.1364/OE.18.027938 21197067

[20] Jain N, Wittmann C, Lydersen L, Wiechers C, Elser D, et al (2011) Device calibration impacts security of quantum key distribution. . Phys. Rev. Lett. 107: 110501 10.1103/PhysRevLett.107.110501 22026652

[21] Lydersen L, Skaar J, Makarov V (2011) Tailored bright illumination attack on distributed-phase-reference protocols. . J. Mod. Opt. 58: 680–685 10.1080/09500340.2011.565889

[22] Lydersen L, Akhlaghi MK, Majedi AH, Skaar J, Makarov V (2011) Controlling a superconducting nanowire single-photon detector using tailored bright illumination. . New J. Phys. 13: 113042 10.1088/1367-2630/13/11/113042

[23] Lydersen L, Makarov V, Skaar J (2011) Comment on “Resilience of gated avalanche photodiodes against bright illumination attacks in quantum cryptography”. Appl. Phys. Lett. 99: 196101 10.1063/1.3658806

[24] Sauge S, Lydersen L, Anisimov A, Skaar J, Makarov V (2011) Controlling an actively-quenched single photon detector with bright light. Opt. Express 19: 23590–23600 10.1364/OE.19.023590 22109239

[25] Lydersen L, Jain N, Wittmann C, Maroy O, Skaar J, et al (2011) Superlinear threshold detectors in quantum cryptography. . Phys. Rev. Lett. 84: 032320 10.1103/PhysRevA.84.032320

[26] Lydersen L, Wiechers C, Wittmann C, Elser D, Skaar J, et al (2010) Avoiding the blinding attack in QKD; REPLY (COMMENT). Nat. Photonics 4: 800–801 10.1038/nphoton.2010.278

[27] Makarov V (2009) Controlling passively quenched single photon detectors by bright light. . New J. Phys. 11: 065003 10.1088/1367-2630/11/6/065003

[28] Kish LB (2006) Totally secure classical communication utilizing Johnson (-like) noise and Kirchooff’s Law. Phy. Lett. A 352: 178–182 10.1016/j.physleta.2005.11.062

[29] Mingesz R, Gingl Z, Kish LB (2008) Johnson (-like) -noise- Kirchhoff-Loop based secure classical communicator characteristics, for ranges of two to two thousand kilometers, via model-line, . Phys. Lett.A 372: 978–984 10.1016/j.physleta.2007.07.086

[30] Kish LB (2006) Protection against the man-in-the-middle-attack for the Kirchhoff-loop-Johnson(-like)-noise cipher and expansion by voltage-based security. Fluctuation and Noise Letters 6: L57–L63 10.1142/S0219477506003148

[31] Kish LB, Horvath T (2009) Notes on recent approaches concerning the Kirchhoff-law-Johnson-noise-based secure key exchange. . Phys. Lett. A 373: 901–904 10.1016/j.physleta.2009.05.077

[32] Kish LB, Saidi O (2008) Unconditionally secure computers, algorithms and hardware. Fluctuation and Noise Letters 8: L95–L98 10.1142/S0219477508004362

[33] Kish LB, Mingesz R (2006) Totally secure classical networks with multipoint telecloning (teleportation) of classical bits through loops with Johnson-like noise. Fluctuation and Noise Letters 6: C9–C21 10.1142/S021947750600332X

[34] Kish LB, Peper F (2012) Information networks secured by the laws of physics. IEICE Transactions on Communications E95-B: 1501–1507.

[35] Horvath T, Kish LB, Scheuer J (2011) Effective privacy amplification for secure classical communications. . Europhys. Lett. 94: 28002 10.1209/0295-5075/94/28002

[36] Hao F (2006) Kish’s key exchange scheme is insecure. IEE Proceedings on Information Society 153: 141–142 10.1049/ip-ifs:20060068

[37] Kish LB (2006) Response to Feng Hao’s paper “Kish’s key exchange scheme is insecure”. Fluctuation and Noise Letters 6: C37–C41 10.1142/S021947750600363X

[38] Scheuer J, Yariv A (2006) A classical key-distribution system based on Johnson (like) noise – How secure? Phys. Lett. A 359: 737–740 10.1016/j.physleta.2006.07.013

[39] Kish LB, Scheuer J (2010) Noise in the wire: The real impact of wire resistance for the Johnson(-like) noise based secure communicator. . Phys. Lett. A 374: 2140–2142 10.1016/j.physleta.2010.03.021

[40] Kish LB (2006) Response to Scheuer-Yariv: “A classical key-distribution system based on Johnson (like) noise – How secure?”. Phys. Lett. A 359: 741–744 10.1016/j.physleta.2006.07.037

[41] Liu PL (2009) A new look at the classical key exchange system based on amplified Johnson noise. . Phys. Lett. A 373: 901–904 10.1016/j.physleta.2009.01.022

[42] Kish LB, Mingesz R, Gingl Z, Granqvist CG (2012) Spectra for the product of Gaussian Noises. Metrology & Measurement Systems 19: 653–658 10.2478/v10178-012-0057-0

[43] Rice SO (1944) Mathematical analysis of random noise. . Bell System Tech. J. 23: 282–332 http://archive.org/details/bstj23-3-282.

[44] Rychlik I (2000) On Some Reliability Applications of Rice's Formula for the Intensity of Level Crossings. Extremes 3: : 331– 348. DOI: 10.1023/A:1017942408501.

[45] Kish LB (2002) End of Moore's Law; Thermal (Noise) Death of Integration in Micro and Nano Electronics. . Phys. Lett. A. 305: 144–149 10.1016/S0375-9601(02)01365-8

[46] Kish LB, Granqvist CG (2012) Electrical Maxwell Demon and Szilard Engine Utilizing Johnson Noise, Measurement, Logic and Control. PLoS ONE 7: e46800 10.1371/journal.pone.0046800 23077525 PMC3471940

[47] Kish LB, Granqvist CG (2012) Energy requirement of control: Comments on Szilard's engine and Maxwell's demon. EPL 98: 68001 10.1209/0295-5075/98/68001

[48] Kish LB (2013) Enhanced secure key exchange systems based on the Johnson-noise scheme. Metrology & Measurement Systems 20: 191–204 10.2478/mms-2013-0017

